# Supplementary material for: Analysis of PPARγ Signaling Activity in Psoriasis
Source: Int J Mol Sci. 2021 Aug 10;22(16):8603. doi: 10.3390/ijms22168603 (PMC8395241; doi:10.3390/ijms22168603)
Supplement: Supplementary file 1 [file ijms-22-08603-s001.zip › Supplemental materials_Analysis of PPARg signaling activity in psoriasis/Pathway models/Models images and html files/PPARG inhibits phenotypes assotiated with psoriasis/136449.html]

pathological angiogenesis


# Disease pathological angiogenesis

|  |  |
| --- | --- |
| URN | urn:agi-meshdis:Neovascularization%2c%20Pathologic |
| Total Entities | 5 |
| Connectivity | 2357 |
| Name | pathological angiogenesis |

---

|  |  |
| --- | --- |
| ChildConcepts | tumor vasculature |
|  | polypoidal choroidal vasculopathy |
|  | choroidal neovascularization |
|  | corneal neovascularization |
|  | retinal neovascularization |

---

|  |  |
| --- | --- |
| Pathway | PPARG inhibits phenotypes assotiated with psoriasis |
|  | efects selected |
|  | Neighbors of pathological angiogenesis |

---

|  |  |
| --- | --- |
| MedScan ID | 9003665 |

---

|  |  |
| --- | --- |
| Alias | neovascularization pathologic |
|  | Pathologic Neovascularizations |
|  | Pathologic Neovascularization |
|  | neo-vascularizations |
|  | Pathological Angiogeneses |
|  | neovascularizations (pathology) |
|  | disease-associated neovascularization |
|  | Pathological Neovascularizations |
|  | Pathological Neo-vascularization |
|  | abnormal neovascularization |
|  | Pathologic Angiogeneses |
|  | neovascularizations |
|  | Pathological Neo-vascularizations |
|  | Pathologic Angiogenesis |
|  | Pathological Neovascularization |
|  | neo-vascularization |
|  | pathological vascularization |
|  | Pathological Angiogenesis |
|  | neovascularization |
|  | Pathologic Neo-vascularizations |
|  | pathological vascularizations |
|  | neovascularization (pathology) |
|  | Pathologic Neo-vascularization |

---

|  |  |
| --- | --- |
| MeSH ID | D009389 |
|  | M0014620 |

---

|  |  |
| --- | --- |
| MeSH Heading | Neovascularization, Pathologic |

---
